# Global epicardial J wave with unipolar recording in both ventricles in a case of Brugada syndrome: Masked early repolarization syndrome type 3

**DOI:** 10.1016/j.hrcr.2023.09.017

**Published:** 2023-09-29

**Authors:** Daiki Shako, Satoshi Nagase, Kenzaburo Nakajima, Takeshi Aiba, Tetsuji Shinohara, Kengo Kusano

**Affiliations:** ∗ Division of Arrhythmia, Department of Cardiovascular Medicine, National Cerebral and Cardiovascular Center, Suita, Japan; † Department of Advanced Arrhythmia and Translational Medical Science, National Cerebral and Cardiovascular Center, Suita, Japan; ‡ Department of Cardiology and Clinical Examination, Faculty of Medicine, Oita University, Yufu, Japan

**Keywords:** Brugada syndrome, Early repolarization syndrome, J-wave syndrome, Unipolar electrogram, Ablation

**Key Teaching Points**

• Recurrence of ventricular fibrillation (VF) after epicardial ablation in Brugada syndrome (BrS) has been reported. In such cases, the area of ablation may not have been sufficient.
• In this case, although the early repolarization pattern (ERP) in the inferior and lateral leads was unclear on electrocardiogram (ECG), direct epicardial mapping with unipolar recording showed J waves not only in the right ventricular outflow tract (RVOT) but also in the inferior right ventricle (RV) and left ventricle (LV), especially after pilsicainide administration. Accordingly, this case was inherently considered to be early repolarization syndrome (ERS) type 3.
• Additional ablation was performed other than RVOT with reference to unipolar J-wave and bipolar delayed potential. Since then, there has been no recurrence of VF.
• The primary difference between BrS and ERS is in the site of the most affected ventricle; however, it should be difficult to discriminate between the two by ECG alone. Even in BrS patients without inferolateral ERP, it is important to map and ablate the inferior RV and LV as well, especially using the unipolar J wave as an indicator.

## Introduction

Brugada syndrome (BrS) is characterized by right precordial J-ST-segment elevation on electrocardiogram (ECG) and increased risk of sudden cardiac death from ventricular fibrillation (VF).^1^ Several reports have suggested that the early repolarization pattern (ERP) in the inferolateral leads on ECG is a major risk for VF.^2^, ^3^, ^4^ However, inferolateral ERP can be masked by myocardial conduction delay and/or bundle branch block in a remote region.^5^^,^^6^ Although BrS and early repolarization syndrome (ERS) differ with regard to ECG lead location of ERP, they are considered to represent a continuous spectrum of phenotypes, and Antzelevitch and colleagues^7^^,^^8^ proposed the term “J-wave syndrome.” When ERP is recognized globally in the inferior, lateral, and right precordial leads, it is classified as ERS type 3, in which the arrhythmia substrate is widely present in both ventricles. Epicardial catheter ablation mainly on the right ventricular outflow tract (RVOT) can suppress VF episodes in the majority of patients with BrS.^9^ However, some patients show recurrence of VF after ablation,^10^ which might be owing to insufficient elimination of VF substrate. In a recent basic study, it was proposed that epicardial J wave with unipolar recording could guide mapping and ablation of abnormal arrhythmia substrate.^11^ Here, we report the first case of BrS in which epicardial unipolar mapping revealed prominent J wave in RVOT, inferior right ventricle (RV), and left ventricle (LV), although inferolateral ERP on ECG was less evident.

## Case report

**Figure 1:**
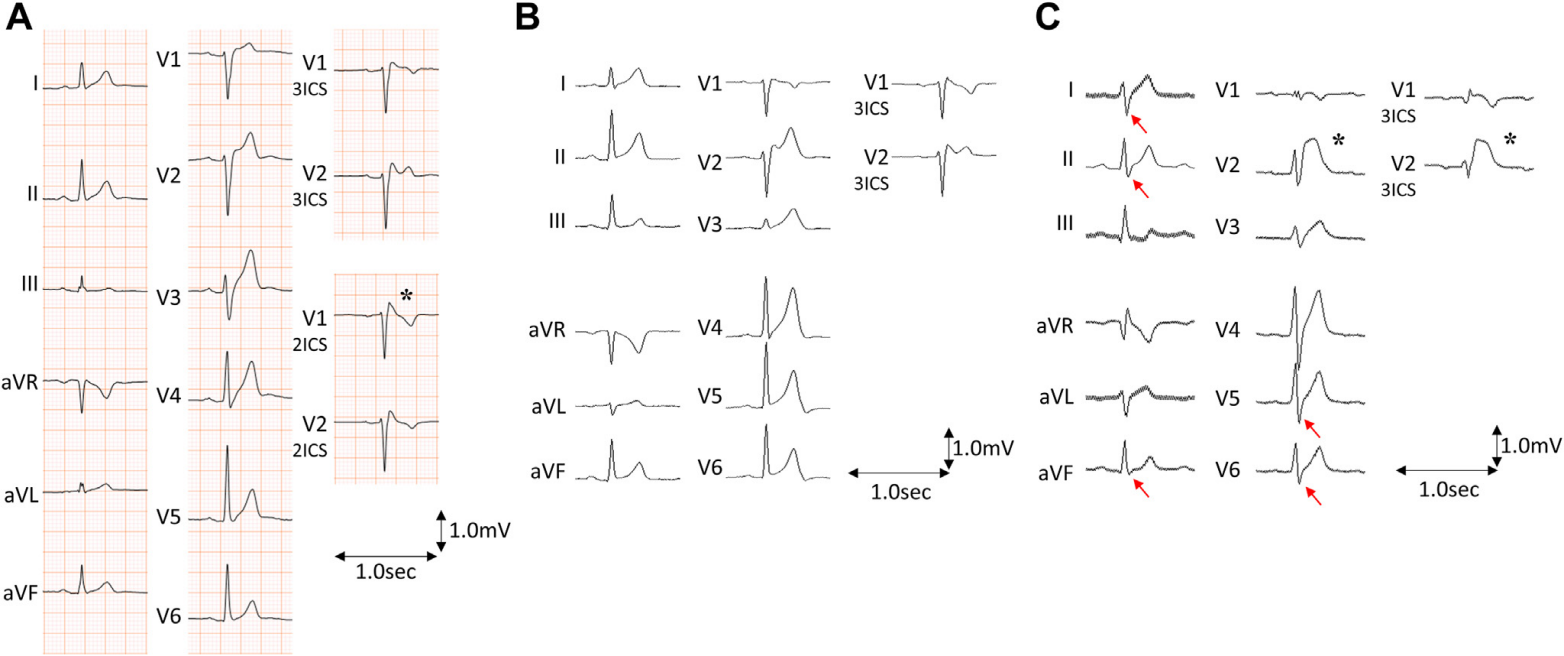
Electrocardiograms (ECGs) **A:** ECG on admission. No obvious early repolarization pattern (ERP) was observed in the inferior and lateral leads. A type 1 Brugada pattern (∗) was recorded in lead V_1_ on the second intercostal space. **B:** Intraoperative ECG before pilsicainide administration. There was no obvious ERP or type 1 Brugada pattern. **C:** Intraoperative ECG after pilsicainide administration. The type 1 pattern appeared in the right precordial leads (∗). However, ERP was completely absent with the appearance of S waves (*red arrows*) in leads I, II, aVF, V_5_, and V_6_. 2ICS = second intercostal space; 3ICS = third intercostal space; ∗ = type 1 Brugada pattern.

The patient was a 32-year-old man with BrS who had been resuscitated from VF; an implantable cardioverter-defibrillator (ICD) had been placed at the age of 21. Because of several VF episodes following ICD implantation, quinidine (300 mg/day) was administered orally, and no recurrence of VF was observed thereafter. However, production of quinidine in Japan was interrupted in 2021, and the supply became insufficient. The patient was switched to a combination of bepridil and cilostazol; however, owing to recurrence of frequent VF episodes, he was admitted to our institution for catheter ablation. ECG revealed spontaneous type 1 Brugada pattern on the right precordial lead with normal QT interval (Figure 1A); echocardiography and coronary computerized tomography showed no abnormalities. Genetic analysis revealed no concerning *SCN5A* gene variants.

**Figure 2:**
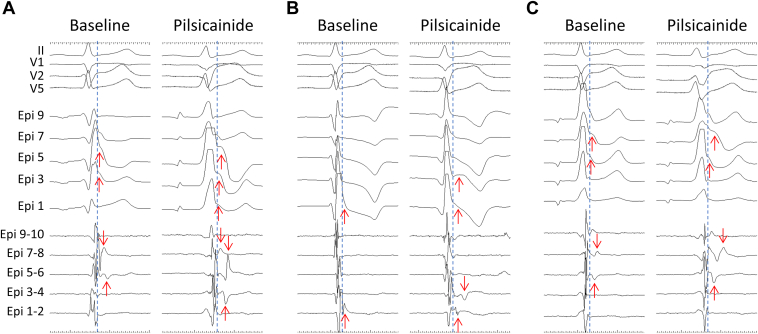
Electrocardiograms and epicardial electrograms. Bipolar (Epi 1-2 to Epi 9-10) and unipolar (Epi 1 to Epi 9) epicardial electrograms on the right ventricular outflow tract (**A**), right ventricular inferior wall (**B**), and left ventricular lateral wall (**C**) under baseline condition (Baseline) and after administration of pilsicainide (Pilsicainide). Red arrows indicate bipolar delayed potential or unipolar J wave.

**Figure 3:**
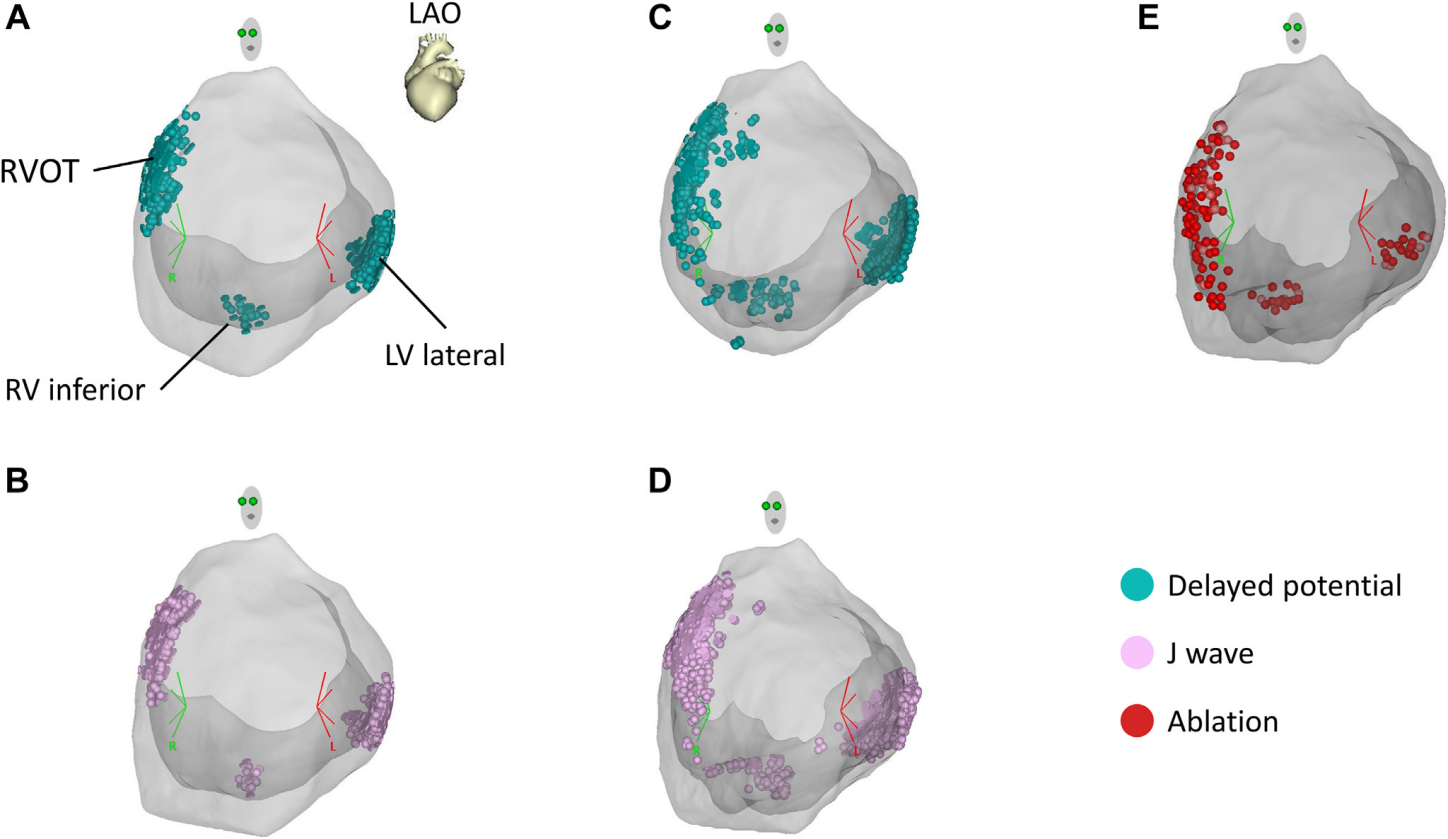
Location of delayed potential (**A, C**), prominent J wave (**B, D**), and radiofrequency application (**E**) plotted on the 3-dimensional mapping system. Images in A and B represent the baseline condition and C and D are after administration of pilsicainide. Delayed potentials, prominent J waves, and ablation sites are plotted with light blue, pink, and red tags, respectively. LV = left ventricle; RV = right ventricle; RVOT = right ventricular outflow tract.

After written informed consent was obtained from the patient, epicardial mapping and ablation were performed under general anesthesia. A 3-dimensional (3D) mapping system (CARTO 3; Biosense Webster, Irvine, CA) and a multipolar mapping catheter (DECANAV; Biosense Webster) were used. An electrode catheter (Inquiry; St. Abbott Laboratories, Chicago, IL) was placed in the inferior vena cava via a femoral vein as an indifferent electrode to record a unipolar electrogram. We recorded local unipolar electrograms with a 0.05- to 100-Hz bandwidth and local bipolar electrograms with a 30- to 250-Hz bandwidth on a digital recording system (LabSystem PRO; Bard Electrophysiology, Lowell, MA), as described previously.^5^^,^^6^^,^^12^ Unipolar electrograms with a 0.05- to 120-Hz bandwidth and bipolar electrograms with a 16- to 500-Hz bandwidth on the 3D mapping system were also recorded. Under baseline conditions, a type 1 pattern and inferolateral ERP were not apparent on the ECG (Figure 1B). Both ventricular epicardial mappings revealed that delayed potentials with bipolar recording were recorded not only in the RVOT but also in the inferior RV and the basal lateral LV (Figures 2 and 3A). Unipolar mapping also revealed that prominent J waves were recorded at the RVOT, inferior RV, and basal lateral LV (Figure 3B). After administration of 25 mg of pilsicainide intravenously, epicardial mapping on both ventricles revealed that the delayed potential with bipolar recording and prominent J wave with unipolar recording were exacerbated as those abnormal regions spread (Figures 2 and 3C and 3D). However, although a type 1 pattern in the right precordial lead was apparent, ERP in the inferior and lateral leads was still not evident (Figure 1C). Rather, with the emergence of the S wave, there was no ERP at all. Radiofrequency ablation of the epicardial substrate with unipolar prominent J-ST elevation and bipolar delayed potential on both ventricles was performed (Figure 3E).

**Supplemental Figure 1:**
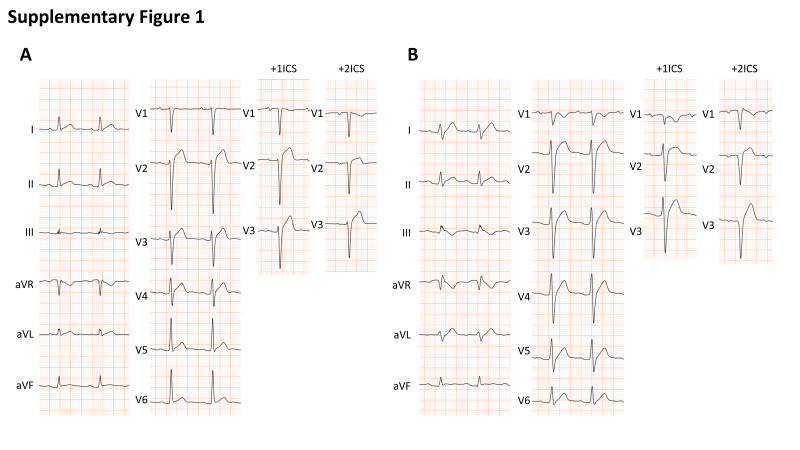
Electrocardiograms after ablation under baseline condition (A) and after administration of pilsicainide (B).

Supplemental Figure 1 shows the ECGs 10 days after ablation; no type 1 pattern was observed even after administration of pilsicainide (1.0 mg/kg). Without any antiarrhythmic drugs, the patient experienced no episodes of appropriate ICD therapy during 14 months of postablation follow-up.

## Discussion

BrS is characterized by J-ST elevation in the right precordial leads, mainly reflecting the RVOT,^13^ and ERP has been reported as an independent predictor of VF occurrence.^2^, ^3^, ^4^ Inferior and lateral ERPs are thought to reflect the inferior ventricular wall and LV lateral wall, respectively.^7^ However, compared with the right precordial leads, inferior and lateral leads are farther from the ventricular epicardium and more likely to be masked by the potential of other regions.^5^ Moreover, conduction delay in remote regions and minor bundle branch block can mask inferior and lateral ERP. Indeed, in the present case, the inferior and lateral ERP was unclear on ECG, but direct epicardial mapping revealed a marked J wave with unipolar recording in the inferior RV and lateral LV, which was particularly prominent after administration of pilsicainide. We speculate that, in inferolateral ECG leads, prominent S wave can mask the ERP that should be present. This phenomenon is similar to that described in our previous reports.^5^^,^^6^

It is unclear whether the spontaneous occurrence of VF in this case was directly associated with epicardial J waves in the inferior RV and lateral LV. However, since this patient with frequent VF episodes had no recurrence for 14 months after ablation without antiarrhythmic drugs, it is likely that treatment other than the RVOT was also important. From the perspective of J-wave mapping and ablation, as proposed by Boukens and colleagues,^11^ detailed mapping should be performed in both ventricular epicardium and ablation should be performed on the potentially arrhythmic substrate as much as possible to eliminate J-wave and delayed potential in J-wave syndrome.

In general, the presence of ERP/J-ST elevation in the inferior, lateral, and right precordial leads is classified as ERS type 3 and it is associated with a high risk of developing spontaneous VF, including VF storms.^7^ In fact, in this case, VF occurred readily when quinidine was discontinued, even 11 years after the first VF occurrence. Interestingly, in this case, the ERP in the inferior and lateral ECG leads was not particularly prominent, but it could be recorded in the epicardium. This case is considered to be highly suggestive in considering the disease concept of J-wave syndrome. It is also expected that patients in whom extensive arrhythmic substrate is reflected as broad epicardial J waves are at higher risk of developing VF; this case was inherently considered to be ERS type 3.

This case report has several limitations. This is a single case report and our observations must be confirmed in more cases. It is unclear whether ablation of sites other than the RVOT was necessary to suppress VF. It is unknown whether J waves or delayed potentials are more critical as targets for ablation, although both were recorded in almost the same area. Cardiac magnetic resonance imaging should also be considered in the future to assess etiology, including the presence of fibrosis.

## Conclusion

In this case of BrS, the ECG showed an indistinct ERP in the inferior and lateral leads, whereas direct epicardial mapping with unipolar recording showed J waves not only in the RVOT but also in the inferior RV and LV. Additional ablations other than RVOT were performed with reference to unipolar J waves and bipolar delayed potentials. Subsequently, no recurrence of VF has been observed for more than 1 year. Even in patients with BrS without inferolateral ERP on ECG, it is worthwhile to map and ablate the inferior RV and LV, particularly using the unipolar J wave as an indicator.

## Disclosures

Dr Satoshi Nagase is affiliated with a department endowed by Japan Medtronic Inc. The remaining authors have nothing to disclose.
